# Cartilage-specific deletion of ephrin-B2 in mice results in early developmental defects and an osteoarthritis-like phenotype during aging in vivo

**DOI:** 10.1186/s13075-016-0965-6

**Published:** 2016-03-15

**Authors:** Gladys Valverde-Franco, Bertrand Lussier, David Hum, Jiangping Wu, Adjia Hamadjida, Numa Dancause, Hassan Fahmi, Mohit Kapoor, Jean-Pierre Pelletier, Johanne Martel-Pelletier

**Affiliations:** Osteoarthritis Research Unit, University of Montreal Hospital Research Centre (CRCHUM), 900 Saint-Denis, R11.412B, Montreal, QC H2X 0A9 Canada; Faculty of Veterinary Medicine, Clinical Science, University of Montreal, Saint-Hyacinthe, QC Canada; Laboratory of Immunology, University of Montreal Hospital Research Centre (CRCHUM), Montreal, QC Canada; Neurosciences Department, Faculty of Medicine, University of Montreal, Montreal, QC Canada; Groupe de recherche sur le sytème nerveux central (GRSNC), Neurosciences Department, Faculty of Medicine, University of Montreal, Montreal, QC Canada; Division of Genetics and Development, Toronto Western Research Institute, University Health Network (UHN) and Department of Surgery, University of Toronto, Toronto, ON Canada

**Keywords:** Ephrin-B2, Bone development, Osteoarthritis, Knockout mouse model

## Abstract

**Background:**

Ephrins and their related receptors have been implicated in some developmental events. We have demonstrated that ephrin-B2 (EFNB2) could play a role in knee joint pathology associated with osteoarthritis (OA). Here, we delineate the in vivo role of EFNB2 in musculoskeletal growth, development, and in OA using a cartilage-specific EFNB2 knockout (EFNB2^Col2^KO) mouse model.

**Methods:**

EFNB2^Col2^KO was generated with Col2a1-Cre transgenic mice. The skeletal development was evaluated using macroscopy, immunohistochemistry, histomorphometry, radiology, densitometry, and micro-computed tomography. Analyses were performed at P0 (birth) and on postnatal days P15, P21, and on 8-week- and 1-year-old mice.

**Results:**

EFNB2^Col2^KO mice exhibited significant reduction in size, weight, length, and in long bones. At P0, the growth plates of EFNB2^Col2^KO mice displayed increased type X collagen, disorganized hyphertrophic zone, and decreased mineralization. At P15, mutant mice demonstrated a significant reduction in VEGF and TRAP at the chondro-osseous junction and a delay in the secondary ossification, including a decrease in bone volume and trabecular thickness. At P21 and 8 weeks old, EFNB2^Col2^KO mice exhibited reduced bone mineral density in the total skeleton, femur and spine. One-year-old EFNB2^Col2^KO mice demonstrated OA phenotypic features in both the knee and hip. By P15, 27 % of the EFNB2^Col2^KO mice developed a hip locomotor phenotype, which further experiments demonstrated reflected the neurological midline abnormality involving the corticospinal tract.

**Conclusion:**

This in vivo study demonstrated, for the first time, that EFNB2 is essential for normal long bone growth and development and its absence leads to a knee and hip OA phenotype in aged mice.

**Electronic supplementary material:**

The online version of this article (doi:10.1186/s13075-016-0965-6) contains supplementary material, which is available to authorized users.

## Background

Erythropoietin-producing hepatocellular receptors (Eph) are the largest family of cell surface receptor tyrosine kinases, representing about 25 % of known receptor tyrosine kinases [1, 2]. There are a total of 15 Ephs classified by sequence homology into subfamilies A and B, but not all are expressed in a given species [3, 4]. Ephs bind to their ephrin (EFN) ligands, which are also cell surface molecules [2]. There are nine EFNs divided into A and B subfamilies. Generally, type A receptors bind preferentially to EFNA, and type B receptors (EphB1-4 and B6) to EFNB, but there are a few exceptions.

The Eph/EFN system was first demonstrated to be essential in the development of neuronal connections, circuit plasticity and repair. Subsequently, their presence and functions have been shown in many organs and tissues in which they were shown to play a role in a number of biological processes [5]. The receptor/ligand EphB4/EFNB2 has been shown to be implicated in bone maintenance and repair [6–8]. However, the role of EFNB2 in skeletal growth and development has never been investigated, likely due to the fact that germ-line mutation of EFNB2 in mice leads to embryonic lethality in homozygous nulls [9, 10].

As both EphB4/EFNB2 are present in the growth plate [8], in vivo EphB4 enhances the process of endochondral ossification bone repair [11], and their presence and activity in adult articular chondrocytes have been demonstrated [12], we further investigated the in vivo role of EFNB2 in skeletal growth and development. To this end, we generated a cartilage-specific EFNB2 knockout (KO) mouse model, using Col2a1-Cre transgenic mice (EFNB2^Col2^KO), as chondrocytes are crucial to bone development.

Moreover, our group previously demonstrated that EFNB2 treatment of human osteoarthritic (OA) chondrocytes positively impacts the abnormal metabolism of these cells [12]. Thus, the present mouse model, in addition to permitting a better understanding of the role of EFNB2 in endochondral bone development, will enable further exploration of the long-term effect of this deletion on knee and hip cartilage in order to substantiate whether this factor could be a new OA therapeutic approach.

## Materials and methods

### Mouse model

Mice were maintained in accordance with the Canadian Council on Animal Care (CCAC) and protocols reviewed and approved by the Institutional Animal Care Committee (CIPA) of the University of Montreal Hospital Centre (CHUM). All mice were kept in a 12-h light/dark cycle. Food and water were available *ad libitum*.

We have previously reported on the generation of EFNB2 floxed (EFNB2^fl/fl^) mice [13]. They were backcrossed with C57BL/6 for 10 generations and then mated with transgenic mice expressing Cre recombinanse driven by type II collagen promoter (Col2a1-Cre) in the C57BL/6 background [14] to obtain mutant mice with a cartilage-specific deletion of EFNB2 (EFNB2^Col2^KO).

The generation and characterization of EFNB2^Col2^KO cartilage conditional mice were as follows. C57BL/6-EFNB2^fl/fl^ mice were mated with C57BL/6 Col2-Cre transgenic mice to generate offspring bearing Col2-Cre and a floxed allele in their germline (genotype: EFNB2^fl/+^, Cre). These mice were backcrossed to homozygote floxed mice in the following cross: EFNB2^fl/+^, Cre X EFNB2^fl/fl^, to generate mice with both alleles inactivated in chondrocytes (genotype: EFNB2^fl/fl^, Cre), and EFNB2^fl/fl^ mice without Cre transgene were used as control mice. Such breeding results in wild type, and heterozygote and homozygote knockout (EFNB2^Col2^KO) mice. The study used the homozygote EFNB2^Col2^KO and the EFNB2^fl/fl^ as controls. The offspring of the breeding animals were genotyped using PCR analysis. The EFNB2^Col2^KO cartilage conditional mice were born at the expected Mendelian frequencies.

**Fig. 1:**
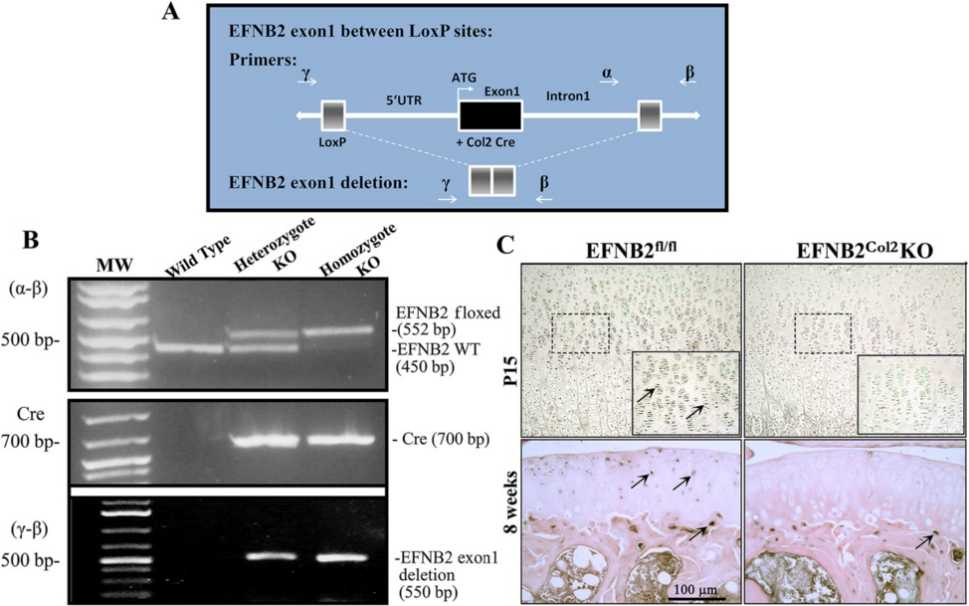
Genetically modified mice harboring a cartilage-specific deletion of ephrin-B2 (*EFNB2*) were generated using the Cre Lox methodology. **a** Schematic representation of the EFNB2 knockout construct and primer design for the LoxP insertion in the EFNB2 gene (α-β) and EFNB2 exon 1 deletion (γ-β). **b** Representative genotyping that detects LoxP insertion in EFNB2 (552 base pairs (bp)), the presence of Cre transgene (700 bp) and EFNB2 exon 1 deletion (550 bp) in heterozygous (n = 8) and homozygous knockout (KO) (n = 8) mice at postnatal day zero (P0) (birth) and their absence in wild type (n = 8) mice assessed by PCR. *MW* molecular weight, *KO* knockout **c** Representative EFNB2 immunohistological staining of the tibial growth plate at P15 (n = 4) counterstained with methyl green, and of tibial cartilage and subchondral bone at 8 weeks old (n = 4) counterstained with hematoxylin and eosin. Immunohistological original magnification × 250 and insets × 400. *Dotted-line boxes* indicate the location of the insets; *scale bar* 100 μm; *arrows* indicate positive-stained cells

The transgenic mouse genotype was determined by PCR analyses of genomic DNA isolated from ear biopsies as previously described [15, 16]. The EFNB2^Col2^KO were identified using the following primers: forward 5′-TCATTTCCCAACCACCGCCAGAAA-3′ and reverse 5′-AGATACCACGCCAGGAGAGCAAAT-3′ for EFNB2; forward 5′-GCATTACCGGTCGATGCAACGAGTGATGAG-3′ and reverse 5′-GAGTGAACGAACCTGGTCGAAATCAGTGCG-3′ for Cre recombinase; and forward 5′-AGATACCACGCCAGGAGAGCAAAT-3′ and reverse 5′-GCGCACGGAGTTGGGTCTCG-3′ for EFNB2 exon 1 deletion. Schematic representation of the EFNB2 knockout construct and primer design are illustrated in Fig. 1a.

The presence of the Cre transgene in postnatal day zero (P0) heterozygous and homozygous knockout mice and its absence in wild type mice were detected by the EFNB2 floxed band at 552 base pairs (bp), the wild type at 450 bp, Cre at 700 bp, and the EFNB2 exon 1 deletion at 550 bp (Fig. 1b). EFNB2^fl/fl^ mice without the Cre transgene were used as control mice. Homozygous mice were compared to control at birth (P0) and on postnatal days P15 and P21, and at 8 weeks and 1 year old.

### Skeletal staining

Skeletal staining was processed in newborn mouse cadavers as described [17].

### Radiographic, bone mineral density and morphometric determinations

High resolution full body radiographic images were obtained using a Kubtec XPERT 80 Digital Cabinet X-ray System with a geometric magnification of × 1 and a resolution of 5 μm (KUB Technologies Inc., Milford, CT, USA). The mouse radiographs were obtained in the ventrodorsal projection with the limbs in abduction (frog-legged position) and a standard ventrodorsal projection with the limbs extended.

The bone mineral density (BMD) was determined using a Lunar PixiMUS 1.46 (GE Lunar Corporation, Madison, WI, USA). The morphometric analyses, including the bone length and hip bone measurements, were performed directly on the acquired images using a calibrated program of the BIOQUANT OSTEO II Image Analysis Software (BIOQUANT Image Analysis Corporation, Nashville, TN, USA).

Other analyses included mouse weight and length at P0, P15 and P21, and long bone measurements of the tibia, femur and humerus at P0 and P15. These measurements were performed directly on freshly dissected bones using digital calipers (model 2071 M; Mitutoyo Corporation, Kawasaki, Japan).

### Micro-computed tomography (μCT)

The left femora of 8-week-old mice were dissected free of tissue, fixed as above and the distal metaphysis scanned with a SKYSCAN 1176 in vivo μCT instrument as described [15]. For the trabecular bone, 100 reconstructed grayscale images were selected from immediately below the tibial and femoral growth plate, and a 3D analysis was used to calculate morphometric parameters, including the bone volume (% bone volume/total volume), trabecular thickness (μm), trabecular number (l/mm) and trabecular separation (mm) at the same threshold with the 3D-Creator software supplied with the instrument.

#### Pelvic and hip acetabular evaluations

Mice at 8 weeks and 1 year old were evaluated for hip dysplasia from the acquired μCT images by measuring the acetabular rim length (ARL, mm), the dorsal acetabular rim angle (DARA, angle degrees) and the acetabular angle (AA, angle degrees) [18]. Values were manually acquired on selected 2D μCT images by two independent observers who were blinded to group allocation. The ARL and the DARA of the right and left acetabula were measured and averaged.

### Histological, histochemical and immunohistochemical analysis

The dissected right long bones were fixed in 4 % paraformaldehyde for 16 h at 4 °C, decalcified in RDO Rapid Decalcifier (Apex Engineering, Plainfield, IL, USA), and embedded in paraffin, as described [15]. Deparaffinized sections (5 μm) were stained with Safranin *O*/fast green (Sigma-Aldrich, Oakville, ON, Canada) and assessed by the BIOQUANT OSTEO II Image Analysis Software. Briefly, a region of 500 μm under the growth plate was selected and the total area was measured. In the same region, the mineralized cartilage matrix (in red) was selected by the color intensity, the area measured, and the ratio calculated.

Immunohistochemical analysis was performed on 5-μm paraffin sections, decalcified in 10 % ethylenediamine tetraacetic acid (EDTA) for 14 days at 4 °C. Briefly, sections were pretreated with 0.25 units/ml of protease-free chondroitinase ABC in phosphate-buffered saline (PBS) (Sigma-Aldrich, St. Louis, MO, USA) for 60 minutes at 37 °C. The specimens were incubated for 18 h at 4 °C with the following primary polyclonal antibodies; rabbit anti-EFNB2 (1:50 dilution; Santa Cruz Biotechnology, Dallas, TX, USA), rabbit anti-type II collagen (1:30 dilution; EMD Millipore, Billerica, MA, USA), mouse anti-proliferating cell nuclear antigen (PCNA) (1:500 dilution; Abcam, Cambridge, MA, USA), rabbit anti-vascular endothelial growth factor (VEGF) (1:1500 dilution; Abcam) and rabbit anti-type X collagen (1:100 dilution; provided by Dr E. Lee, Shriners Hospital for Children, McGill University Hospital Centre, Montreal, QC, Canada) [19]. Each slide was washed three times in PBS (pH 7.4) and incubated with a secondary biotinylated antibody (anti-mouse, or anti-rabbit when appropriate) (Vector Laboratories Inc., Burlingame, CA, USA), then processed using the Vectastain ABC kit (Vector Laboratories) following the manufacturer’s instructions. The color was developed with 3,3’-diaminobenzidine (DAB) containing hydrogen peroxide, and slides were counterstained with methyl green.

Control procedures were performed according to the same experimental protocol as follows: 1) omission of the primary antibody and 2) substitution of the primary antibody with a nonspecific immunoglobulin from the same host as the primary antibody (Santa Cruz Biotechnology).

Tartrate resistant acid phosphatase (TRAP) detection was performed on paraffin sections, decalcified in 10 % EDTA for 14 days at 4 °C. Sections were stained for enzyme activity and processed as described [20] and counterstaining was performed with 0.4 % methyl green. Negative staining was performed without substrate and for all antibodies IgG controls displayed only background staining.

For the calcium deposition, freshly dissected left-side long bones were fixed as above and embedded in glycidyl methacrylate (GMA) plastic. Sections (2 μm) were stained using the von Kossa method with 5 % silver nitrate for 30 minutes under ultraviolet light, and with 0.2 % toluidine blue to determine trabecular bone thickness and bone volume. Histomorphometric data were obtained using BIOQUANT OSTEO II Image Analysis software.

To determine the number of TRAP-, VEGF- and type X collagen-positive cells the chondro-osseous junction area comprising the hypertrophic chondrocyte zone and the cartilage bone junction as the upper and lower limits was evaluated. Hence, for type X collagen, the area analyzed comprised a box of 300 × 100 μm in the hypertrophic chondrocyte zone close to the chondro**-**osseous junction. The staining intensity of the total hypertrophic zone was assessed on one section/specimen using a Leitz Diaplan microscope (Leica Microsystems) connected to BIOQUANT OSTEO II Image Analysis software [15].

### Knee and hip cartilage evaluation

At one year old, the knee and hip joints were evaluated. Sagittal sections (5 μm) from the mid-medial tibiofemoral joint were stained with Safranin *O*/fast green as described [15]. Cartilage was assessed using the Osteoarthritis Research Society International (OARSI) scoring system (knee) [21] and the modified Mankin system (hip) [22]. For the synovial membrane, histomorphometric quantitative analysis of the anterior synovial membrane thickness was performed as described [16]. Images were captured at × 63 with a Leitz Diaplan microscope coupled to a personal computer, and histomorphometric data determined with BIOQUANT OSTEO II Image Analysis Software; data are expressed as μm. The anterior synovial membrane lining hyperplasia was graded on a scale of 0–2, where 0 = absence, 1 = hyperplasia of lining <50 % of the surface, and 2 = hyperplasia of lining >50 % of the surface, as described [23].

For both the cartilage and the synovial membrane, two independent observers graded the severity of the tissue, blinded to group allocation. Three sections were made from each block, each slide was examined, and the final score was a consensus between the two observations.

### Tracing of the corticospinal tract

#### Surgical procedures and tracer injections

Surgical procedures and tracer injections were performed on 6-week-old EFNB2^Col2^KO and control mice anesthetized with a mixture of ketamine (70 mg/kg IP, Ketalar; Pfizer, New York, NY, USA) and xylazine (3 mg/kg IP; Sigma-Aldrich). Mice were placed in a stereotaxic frame and transitioned to an inhalation anesthetic, isoflurane (approximately 2 %) in 100 % oxygen, delivered via a custom-made facial mask as described [24]. After the EFNB2^Col2^KO and control mice were anesthetized, two small holes were made in the skull bone overlying the left primary motor cortex, based on stereotaxic coordinates (0.0, +1 mm anteroposterior, +1.5 mm mediolateral to the bregma). In each hole, 1 μl of the anterograde tracer biotinylated dextran amine (BDA; molecular weight (MW) 10,000, 5 % in saline solution; Invitrogen) was pressure injected with a Hamilton syringe and a microsyringe pump controller (Harvard Apparatus, Holliston, MA, USA). Tracer (1 μl) was injected in three boluses at different depths to create a column that labeled all layers of the gray matter. First, 500 nl was injected at a depth of 2,500 μm from the top of the skull. After a 2-minute rest period to favor the absorption of the tracer by the tissue, a second bolus of 300 nl was injected at 2,000 μm, again followed by a 2-minute rest period. A third bolus of 200 nl was injected at 1,500 μm, followed by a 5-minute rest period. Following injections into the two holes, mice received buprenorphine hydrochloride (0.05 mg/ml IP, Temgesic) and their recovery was monitored during a period of 2 h post surgery. They were then returned to their home cage and given access to food and water *ad libitum*. The mice were sacrificed 14 days after the injections of BDA. They were transcardially perfused with 0.1 M PBS, followed by 4 % paraformaldehyde in 0.1 M PBS. The cervical spinal cords were dissected and post fixed (20 % sucrose, 4 % paraformaldehyde solution in 0.1 M PBS for 2 h; 20 % sucrose, 2 % dimethyl sulfoxide (DMSO) in 0.1 M PBS for 24 h; and 20 % sucrose for 48 h or until the tissues sank). The cervical cord specimens (C4–C7) were quickly frozen at –55 °C with methyl butane, embedded with optimum cutting temperature compound (OCT, Tissue-Tek, Sakura Finetek USA Inc., Torrance, CA, USA) and frozen at –80 °C until sectioning. Transverse sections between C4 and C7 were cut with a cryostat (20 μm thickness). One out of three sections was stained for BDA and used to quantify the number of synaptic boutons.

#### Tissue processing

Tissue processing, in which the BDA staining was revealed, was carried out according to the protocol of Dancause et al. [25]. In brief, after sectioning, the tissues were rinsed in cold 0.05 M potassium phosphate buffer in saline solution (KPBS), treated with 0.4 % Triton X-100 in 0.05 M KPBS and rinsed again in 0.05 M KPBS. They were then incubated overnight in avidin-biotin (ABC) solution (two drops of solution A and B per 5 ml of 0.05 KPBS; Vectastain Elite ABC kit; Vector Laboratories, Burlingame, CA, USA). The following day, following four rinses with 0.1 M KPBS, the tissue was incubated in fresh 0.05 % DAB 0.015 % H_2_O_2_ in KPBS solution for 5–10 minutes. After three additional rinses in 0.1 M KPBS, the sections were mounted on subbed slides and dried overnight. DAB staining was intensified the following day. Sections were dehydrated in ascending alcohol solutions, transferred to xylene, rehydrated and incubated in a 1.42 % AgNO_3_ solution for 1 h at 56 °C. They were then passed through H_2_O (15 minutes), 0.2 % HAuCl_4_ (10 minutes), 5 % Na_2_S_2_O_3_ (5 minutes) and H_2_O (15 minutes). Finally, they were dehydrated again followed by xylene and coverslipped the next day. Other sections were either Nissl stained with Cresyl violet to reveal spinal cord architecture (1/3) or kept for future staining (1/3).

#### Image capture and quantitative analysis of BDA

Image capture and quantitative analysis of BDA was performed as described [26]. In brief, the tissue was examined using a BX51 light microscope (Olympus, Tokyo, Japan). Photographs were digitally captured using an MBF CX9000 digital camera (MicroBrightField, Colchester, VT, USA) with a resolution of 1600 × 1200 active pixels and images imported to Adobe Photoshop CS5. A neuroanatomical reconstruction system, consisting of a computer-interfaced motorized stage mounted on a microscope and associated software (Neurolucida; MicroBrightField), was used to reconstruct sections stained for BDA and to quantify the number of labeled boutons at the C4–C7 levels. The most rostral section of the block to be reconstructed was randomly chosen. Then, one section out of every six BDA-stained sections was reconstructed up to a total of ten sections. A varicosity was considered to be a terminal bouton if it appeared as a small, darkly labeled sphere contacting a small fiber as described [26]. To quantify the number of labeled boutons within each hemisphere of the spinal cord, we examined the tissue using a grid pattern (100 × 100 μm) overlaid on the section image. If at least two synaptic boutons were located within a square of the grid, a marker was placed in the center of the square. Following inspection of the entire gray matter, the numbers of positive squares in each hemisphere were compared.

### Statistical analysis

Values are expressed as mean ± standard error of the mean (SEM). Statistical analysis was performed using the Mann–Whitney *U* test (GraphPad Prism software, GraphPad, San Diego, CA, USA).

## Results

### Characterization of EFNB2^Col2^KO cartilage conditional mice

The genotyping demonstrated the presence of the Cre transgene in P0 heterozygous and homozygous knockout mice and its absence in wild type mice (Fig. 1b). To confirm the cartilage specific deletion, immunohistochemical analysis using a specific EFNB2 antibody was performed on the tibiae at P15 and at 8 weeks old. EFNB2 was present in the cartilage of the control (EFNB2^fl/fl^) and absent in the EFNB2^Col2^KO (Fig. 1c). Of note, EFNB2 was present in the subchondral bone of both the EFNB2^fl/fl^ and EFNB2^Col2^KO mice (Fig. 1c).

### Reduction in growth of EFNB2^Col2^KO mice

**Fig. 2:**
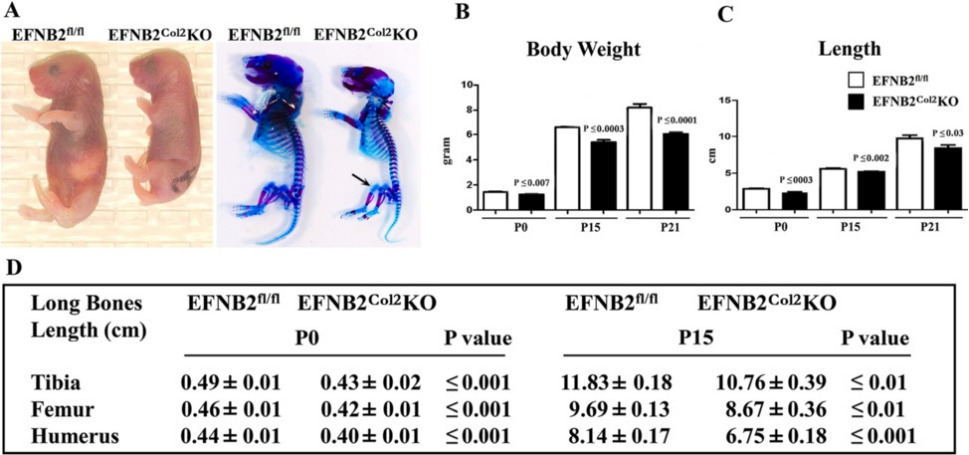
Macroscopy and morphological assessments of male ephrin-B2 (*EFNB2*)^fl/fl^ and EFNB2^Col2^knockout (*KO*) cartilage conditional mice. **a** Representative mascroscopic and histochemical results of EFNB2^fl/fl^ (n = 6) and EFNB2^Col2^KO cartilage conditional (n = 6) mice at postnatal day zero (*P0*) (birth) showing that the latter displayed reduced growth and skeletal development. *Arrow* indicates thinner stained region in the EFNB2^Col2^KO. Histograms show body weight (**b**) and length (**c**) of EFNB2^fl/fl^ and EFNB2^Col2^KO cartilage conditional mice at P0 (n = 8 and n = 9, respectively), P15 (n = 10 and n = 8) and P21 (n = 13 and n = 11). **d** Morphometric analysis of long bone length of EFNB2^fl/fl^ and EFNB2^Col2^KO cartilage conditional mice at P0 and P15 (n = 8–13). Data are expressed as mean ± standard error of the mean and *P* values were determined by Mann–Whitney *U* test

In newborn litters EFNB2^Col2^KO were smaller in size compared to control mice (Fig. 2a). The delayed pattern was observed in both genders but only data on male animals are presented in this article.

Skeletal staining confirmed the reduced growth in the homozygote EFNB2^Col2^KO mice compared to controls at birth, and there was less skeletal staining in some regions of the limbs of mutant mice than in the control mice (Fig. 2a).

Morphometric analysis at P0, P15 and P21 showed that the EFNB2^Col2^KO mice displayed a significant reduction in body weight and length (Fig. 2b, c) when compared with control mice. In addition, on measurement of individual long bones at P0 and P15 there was a significant reduction in the long bone length (tibia, femur and humerus) in the EFNB2^Col2^KO mice (Fig. 2d). At P21, 8 weeks and 1 year, the EFNB2^Col2^KO mice had significantly smaller tibiae (*P* ≤0.03, *P* ≤0.01, and *P* ≤0.02, respectively) and femora (*P* ≤0.03, *P* ≤0.03, and *P* ≤0.04) compared with their controls.

### Impaired angiogenesis and delayed cartilage resorption in EFNB2^Col2^KO mice

As bone length is determined in part by the activity of the growth plate during endochondral bone formation, we further examined the growth plate morphology in the EFNB2^Col2^KO mice. At P0, EFNB2^Col2^KO mice had abnormalities at the growth plates of appendicular bones (tibia, femur and humerus); there was a disorganized hypertrophic cartilage zone with no difference in the reserve or proliferating zones.

**Fig. 3:**
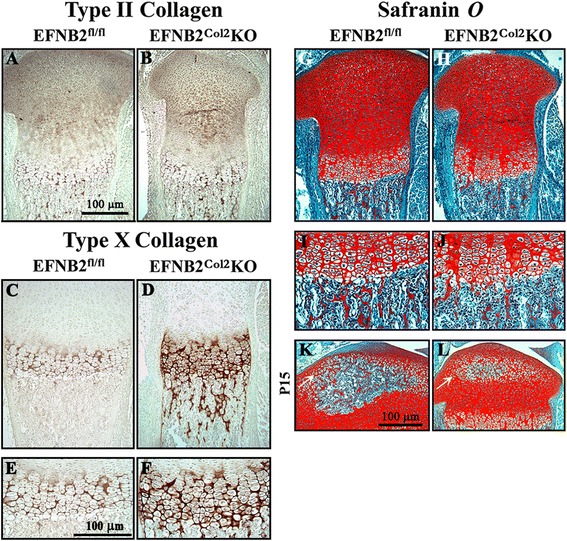
Immunohistochemical localization of type II collagen, type X collagen and Safranin *O* in epiphyseal cartilage. Representative immunohistological sections of ephrin-B2 (*EFNB*2)^fl/fl^ (n = 7) and EFNB2^Col2^knockout (*KO*) cartilage conditional (n = 7) mice at postnatal day zero (P0) (birth) for type II collagen (**a**-**b**), type X collagen (**c**-**f**) and Safranin *O* (**g**-**l**) of the entire epiphyseal cartilage. **e**, **f** Higher magnification of **c** and **d**. **i**, **j** Higher magnification of **g** and **h**. The secondary center of ossification at P15 is indicated by *white arrows* (**k** and **l**). **a**-**f** Slides were counterstained with methyl green. Original magnification × 250 (**a**-**d**, **g**, **h**, **k**, **l**) and × 400 (**e**, **f**, **i**, **j**). *Scale bars* at 100 μm (**a**, **e**, **k**)

**Fig. 4:**
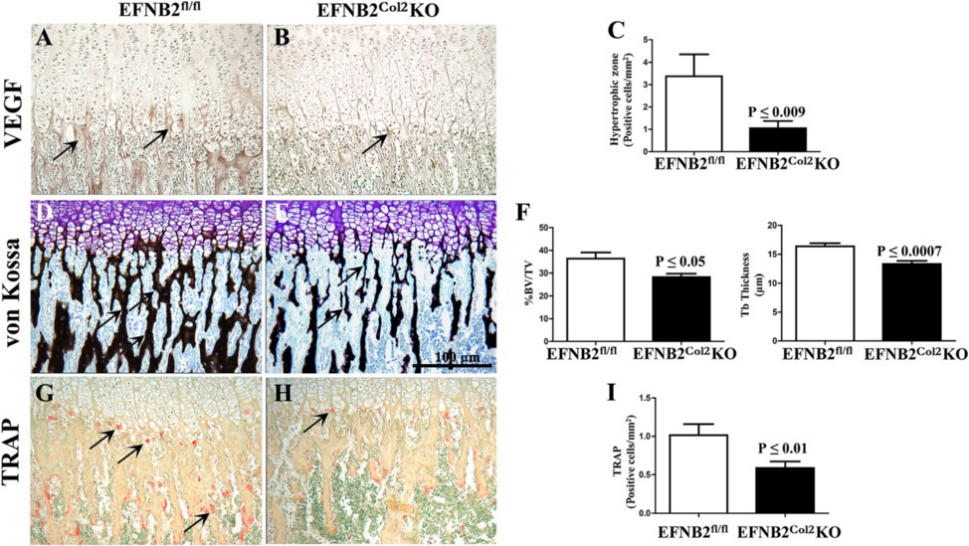
Impaired vascularization of ephrin-B2 (*EFNB2*)^Col2^knockout (*KO*) cartilage conditional mice at postnatal day P15. Representative immunohistochemical and histochemical sections of proximal tibia, and histograms of EFNB2^fl/fl^ (n = 7) and EFNB2^Col2^KO cartilage conditional (n = 7) mice at P15 for vascular endothelial growth factor (*VEGF*) (**a-c**)), von Kossa (**d**-**f**) and tartrate resistant alkaline phosphatase (*TRAP*) (**g**-**i**). Sections were counterstained with methyl green. Original magnification × 400. **e** *Scale bar* at 100 μm. **f** *%BV/TV* % bone volume/tissue volume, *Tb Thickness* trabecular thickness. *Arrows* indicate positive staining (**a**, **b**, **g**, **h**) and trabecular thickness (**d**, **e**). Data are expressed as the mean ± standard error of the mean and *P* values were determined using the Mann–Whitney *U* test

Type II collagen is the principal collagen laid down by proliferating, non-hypertrophic chondrocytes, whereas type X collagen production is restricted to hypertrophic cells in the epiphyseal cartilage. There were no differences in type II collagen (Fig. 3a, b) or the proliferation marker, PCNA (data not shown), implying that early stages of chondrocyte development were not affected by the lack of EFNB2. However, type X collagen immunostaining displayed disorganized hypertrophic chondrocyte columns (Fig. 3c-f) with increased staining (*P* <0.03) accompanied by a decrease in mineralized cartilage matrix (*P* <0.007) (Fig. 3g-j) at the chondro-osseus junction, which was also observed at P15 (*P* <0.03). In addition, P15 EFNB2^Col2^KO mice had a delay at the secondary center of ossification (Fig. 3k, l) and significantly decreased VEGF staining (Fig. 4a-c). The disturbed vascularization was associated with alterations in bone mineralization; the percentage of bone volume/tissue volume and the trabecular thickness were also significantly reduced in the EFNB2^Col2^KO mice compared to controls (Fig. 4d-f).

The terminal stage of hypertrophic chondrocyte development is associated with invasion and resorption of the calcified cartilage core. Given the delay in calcification in the EFNB2^Col2^KO mice, we further investigated whether the lack of EFNB2 disturbed the cartilage resorption process. The recruitment of and invasion by TRAP-positive cells was significantly reduced in the EFNB2^Col2^KO mice compared to controls (Fig. 4g-i).

### Postnatal bone abnormalities in EFNB2^Col2^KO mice

**Fig. 5:**
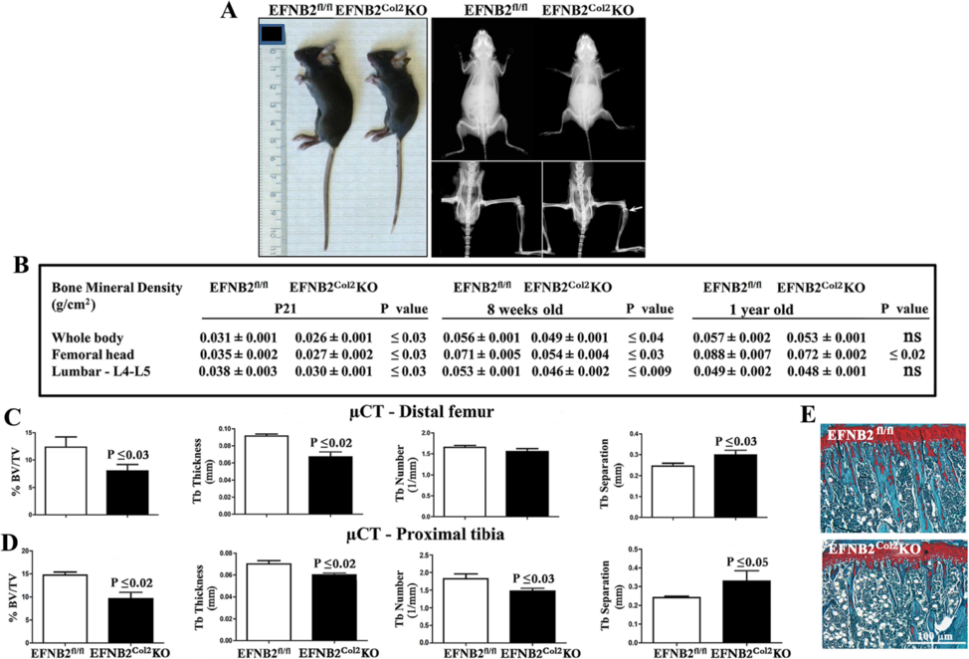
Bone abnormalities of the ephrin-B2 (*EFNB2*)^Col2^knockout (*KO*) cartilage conditional mice. **a** Representative macroscopic and radiographic EFNB2^fl/fl^ (n = 6) and EFNB2^Col2^KO cartilage conditional (n = 6) mice at postnatal day P21. In the radiographs, the lower portion of EFNB2^Col2^KO cartilage conditional mice shows osteopenia (*arrow*) compared to EFNB2^fl/fl^ mice. **b** Bone mineral density measurements of EFNB2^fl/fl^ and EFNB2^Col2^KO cartilage conditional mice at P21, 8 weeks and 1 year (n = 8–10 for each category). Micro-computed tomography (μCT) analysis of the distal femur (**c**) and proximal tibia (**d**) (n = 8) at 8 weeks. **e** Representative histological sections of the proximal tibia stained with Safranin *O*; original magnification × 400, *scale bar* at 100 μm. *% BV/TV*o % bone volume/tissue volume, *Tb* trabecular thickness. Data are expressed as mean ± standard error of the mean and the *P* values were determined by Mann–Whitney *U* test

Macroscopic and radiographic assessments at P21 showed reduced body size of EFNB2^Col2^KO mice (Fig. 5a). Although no bone deformities were found in the EFNB2^Col2^KO mice, osteopenia was observed in the long bones (Fig. 5a). BMD analysis (Fig. 5b) showed that EFNB2^Col2^KO mice had a significant reduction in the BMD of the whole body, femoral head, and lumbar vertebrae L4 and L5 at P21 and 8 weeks. At 1 year, although BMD values were lower for each of the bones studied, only for the femoral head BMD was statistically significant.

μCT of the femur and tibia in the 8-week-old EFNB2^Col2^KO mice showed a statistically significant reduction in the mineralized tissue (about 35 %, as evaluated by the % bone volume/tissue volume) (Fig. 5c, d). Trabecular number and thickness in the proximal tibia were also significantly decreased with an increase in trabecular separation compared to control (Fig. 5d). Similar differences were observed in the distal femur with the exception of trabecular number (Fig. 5c). Histological sections of the proximal tibia confirmed μCT data revealing thinner trabeculae and an increase in the trabecular separation (Fig. 5e). This decrease in mineralized tissue in the EFNB2^Col2^KO could be the result of improper cartilage matrix degradation caused by EFNB2 deficiency in late hypertrophic chondrocytes.

### Osteoarthritis (OA) phenotypic features in aged EFNB2^Col2^KO mice

**Fig. 6:**
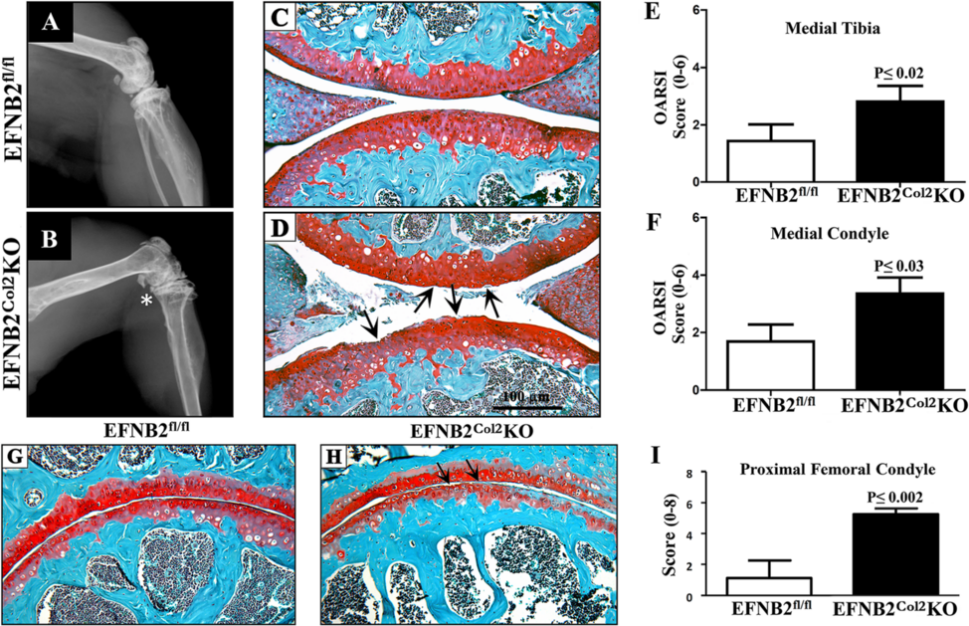
Osteoarthritis features in ephrin-B2 (*EFNB2*)^Col2^knockout (*KO*) cartilage conditional mice at 1 year old. Representative radiographs (**a**, **b**) and histological sections (**c**, **d**) of the knee joint of EFNB2^fl/fl^ (n = 8) and EFNB2^Col2^KO cartilage conditional (n = 8) mice stained with Safranin *O*/fast green. Histograms show Osteoarthritis Research Society International (*OARSI*) scoring of the medial tibia (**e**) and medial condyle (**f**). **g**, **h** Histological sections of the hip joint stained with Safranin *O*/fast green. **i** Hip scoring according to the modified Mankin system. *Decrease in the joint space (**b**). *Scale bar* at 100 μm (**d**). *Arrows* indicate cartilage degradation (**d**, **h**). Histological sections at original magnification × 100. Data are expressed as the mean ± standard error of the mean and *P* values were determined by Mann–Whitney *U* test

One-year-old EFNB2^Col2^KO mice demonstrated OA phenotypic features associated with cartilage degeneration in both the knee and hip (Fig. 6). Radiologically, EFNB2^Col2^KO mice exhibited a collapse of the joint with decreased joint space (Fig. 6a, b). Histological analysis of both the knee (medial tibia and condyle) and the proximal femoral condyle showed that EFNB2^Col2^KO mice had increased cartilage degradation with a loss of Safranin *O*, reduced cellularity and thinning of the cartilage (Fig. 6c-i). As synovial membrane also demonstrated alterations during the OA process, we further assessed the effects of cartilage-specific EFNB2 deletion on this tissue in 1-year-old mice. The synovial thickness was significantly greater in the knee in the EFNB2^Col2^KO mice (43.4 μm ± 5.6) than in the control mice (113.7 μm ± 27.2) (*P* ≤0.02) and in the hip (86.9 μm ± 5.9 compared to 143.3 μm ± 18.5, respectively) (*P* ≤0.03). There were no significant differences in the synovial lining cells between EFNB2^Col2^KO and the control mice.

### Some EFNB2^Col2^KO mice exhibit a locomotor phenotype related to an abnormal corticospinal tract

**Fig. 7:**
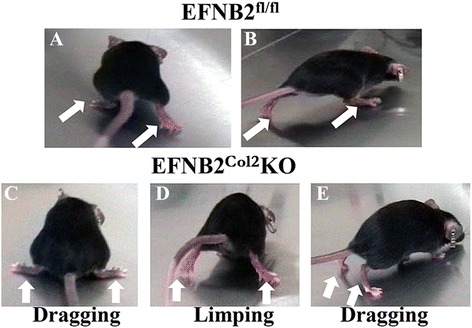
Some ephrin-B2 (*EFNB2*)^Col2^knockout (*KO*) cartilage conditional mice exhibited a locomotor phenotype. EFNB2^fl/fl^ (**a**, **b**) and about 27 % of the EFNB2^Col2^KO cartilage conditional mice (**c**-**e**) demonstrated a locomotor phenotype consisting of dragging and limping as soon as they started to walk. Additional movie files show the locomotor phenotype in more detail (see Additional file 1 (EFNB2^fl/fl^) and Additional file 2 (EFNB2^Col2^KO))

A surprising feature of the EFNB2^Col2^KO mice was a locomotor phenotype observed in about 27 % of this mouse population, which appeared as soon as the mice started to walk at 2–3 weeks of age. It consisted of a lack of unilateral hip motor control yielding a simultaneous movement of the right and left limbs, resulting in a hopping gait (Fig. 7), unlike the alternate step gait displayed by control mice. Additional movie files show this in more detail (see Additional file 1 and Additional file 2).

**Fig. 8:**
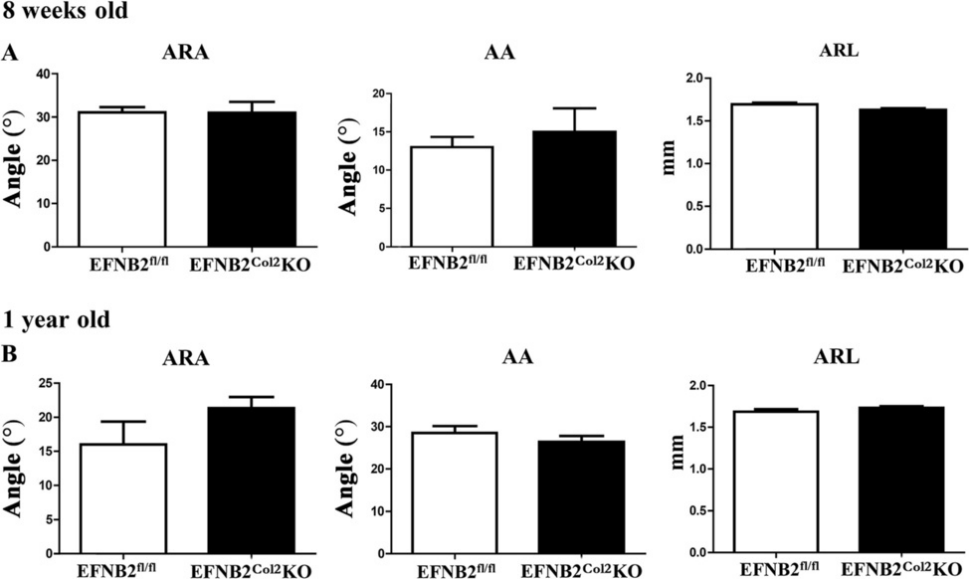
Micro-computed tomography evaluations of the proximal femoral head of the acetabular rim angle, acetabular angle and acetabular rim length of the ephrin-B2 (*EFNB2*)^Col2^knockout (*KO*) mice with a locomotor phenotype at both 8 weeks of age and 1 year. **a** Acetabular rim angle (*ARA*), acetabular angle (*AA*) and acetabular rim length (*ARL*) of EFNB2^Col2^KO and EFNB2^fl/fl^ (n = 5 and 4, respectively) at 8 weeks and **b** at 1 year (n = 5 for both groups). Data are expressed as the mean ± standard error of the mean and the *P* values were determined by Mann–Whitney *U* test

The EFNB2^Col2^KO long bone developmental abnormalities could not account for the observed locomotor phenotype. However, the EFNB2^Col2^KO mice had a significantly (*P* ≤0.01) smaller pelvic bone at 8 weeks compared to controls, but not at 1 year (data not shown). We then further investigated the possibility of abnormal development of the hip joint. μCT of the EFNB2^Col2^KO proximal femoral head of the acetabular rim angle, acetabular angle and acetabular rim length of 8-week-old and 1-year-old mice did not differ from controls (Fig. 8), suggesting that the EFNB2^Col2^KO mice did not have any hip joint abnormalities that could explain the locomotor phenotype.

The above finding indicates that a cause other than the hip development is responsible for the EFNB2^Col2^KO locomotor phenotype. A search of the literature revealed that this locomotor phenotype resembles those reported for mice lacking the EFNB3 ligand or its receptor EphA4 [27–30]. This defect has been related to a critical role played by these factors in establishing corticospinal projection, in which the lack of unilateral control results from an embryonic midline abnormality involving the corticospinal tract [28, 29]. This points to the possibility that the EFNB2^Col2^KO mice might have had abnormalities in the cortical tract even though the deletion was conditional to type II collagen.

**Fig. 9:**
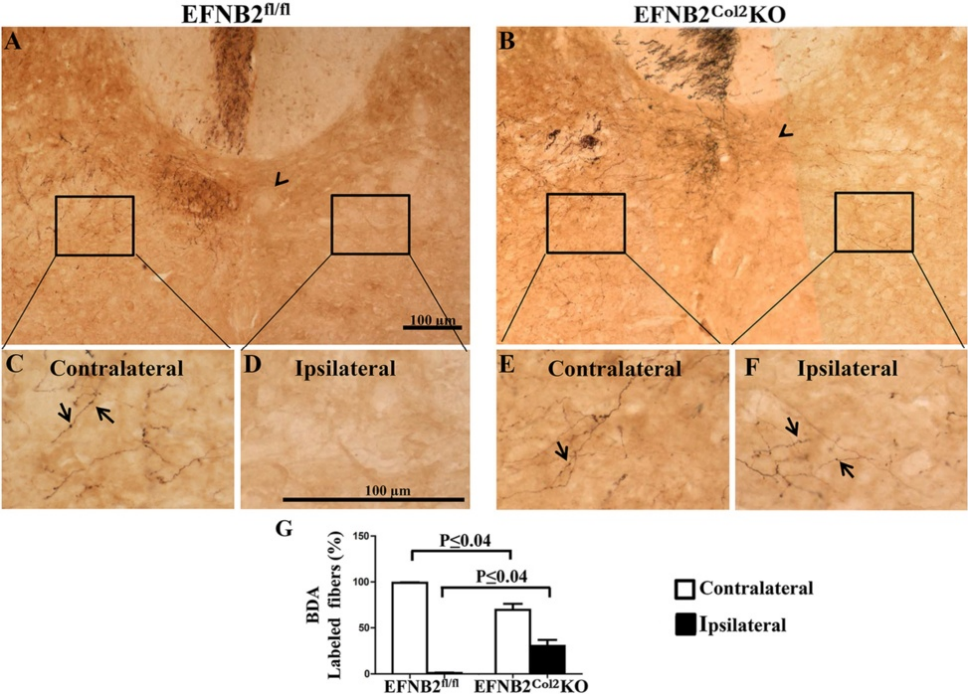
Locomotor phenotype of some ephrin-B2 (*EFNB2*)^Col2^knockout (*KO*) cartilage conditional mice is related to an abnormal corticospinal tract. **a**-**f** Brain tracing experiments to visualize the corticospinal path with biotinylated dextran amines (*BDA*) performed in 6-week-old EFNB2^fl/fl^ (n = 2) and EFNB2^Col2^KO cartilage conditional (n = 3) mice. Representative photomicrographs of the cervical spinal cord of EFNB2^fl/fl^ (**a**, **c**, **d**) and EFNB2^Col2^KO cartilage conditional mice (**b**, **e**, **f**) at the level of the fifth cervical vertebra. **g** Percentage of BDA-labeled fibers in the contralateral and ipsilateral hemispheres. *Arrowheads* indicate the injection site (**a**, **b**) and labeled fibers (**c**-**f**). *Scale bars* at 100 μm (**a**, **d**). Data are expressed as the mean ± standard error of the mean and the *P* values were determined by Mann–Whitney *U* test

We then looked at the corticospinal tract axons of the mice with the locomotor phenotype. To visualize the path taken by the corticospinal tract axons, we performed anterograde axon tracing experiments by injecting a tracer into one side of the motor cortex and observing the terminal projections in the spinal cord. Compared to the control mice, there was a dramatic difference in the path of EFNB2^Col2^KO mouse corticospinal axons on entering the spinal dorsal gray matter. In the control mice, projections into the gray matter remained confined to the contralateral gray matter, never crossing the midline, as shown in cervical sections (Fig. 9a, c, d). In contrast, the corticospinal tract projections of EFNB2^Col2^KO mice clearly crossed over the midline into the ipsilateral gray matter (Fig. 9b, e, f). Indeed, in the control mice, a high level of labeled fibers was visible in the hemisphere contralateral to the injection site, which is close to the midline (Fig. 9a, c), whereas few labeled fibers were found in the ipsilateral hemisphere (Fig. 9d). In contrast, the EFNB2^Col2^KO mice exhibited labeled fibers in both hemispheres (contralateral and ipsilateral) (Fig. 9b, e, f), which, compared to the control mice, were significantly fewer in the contralateral hemisphere but more numerous in the ipsilateral hemisphere (Fig. 9g). These neuroanatomical findings suggest that the locomotive phenotype was due to a neurological defect in corticospinal projections.

## Discussion

In this study, we investigated the role of EFNB2 during endochondral bone development and its effect on adult articular cartilage integrity, by generating cartilage-specific EFNB2 KO mice. This model was chosen because global deletion of EFNB2 in mice leads to embryonic lethality [9, 10]. This study is the first to show that EFNB2 is essential for postnatal skeletal growth, as its absence in growth plates resulted in a delay of long bone growth including delayed primary and secondary centers of ossification, accompanied by increased mineralized cartilage, delayed vascular invasion, and a reduction in TRAP-positive cells, bone density and formation of bone trabeculae. Furthermore, the cartilage-specific ablation of EFNB2 also led to spontaneous features of OA associated with a decrease in joint space, cartilage degeneration and synovial membrane alterations in both knee and hip joints. Importantly, the latter was not related to the locomotor phenotype, as this was observed in only 27 % of EFNB2 KO mice, and all mice had the OA defects. Collectively, these data reinforce the hypothesis that EFNB2 plays an important role in cartilage growth and maintenance; its absence impairs endochondral ossification and cartilage development and predisposes the joint to degeneration resembling OA.

Endochondral bone formation and growth, during which the cartilage provides a template on which bone is laid down, are critically dependent on chondrocyte metabolism and this process involves highly organized cartilaginous growth plates [31–33]. Here, we demonstrated that the delayed ossification and growth plate alteration observed in the EFNB2^Col2^KO mice appears not to be due to abnormal growth and differentiation of chondrocytes, as shown by the normal pattern of PCNA and type II collagen, markers of proliferation and chondrocyte differentiation [34]. Thus, EFNB2 seems to have no significant influence on either the influx of resting zone cells or on the mitogenic activity of proliferative cells in the growth plate. However, the increased type X collagen observed in the growth plates of EFNB2^Col2^KO mice suggests an abnormal chondrocyte metabolism that could lead to shortening of the long bones, as this collagen facilitates endochondral ossification by regulating matrix mineralization and compartmentalizing matrix components [35, 36]. As our data showed that EFNB2 does not appear to be involved in the early stages of chondrocyte development, but affects the hypertrophic cells in the epiphyseal cartilage, it would be of interest to conduct a further study in which the loss of EFNB2 is specifically restricted to the hypertrophic chondrocytes of the growth plate cartilage, using the Col10a1 promoter.

A mechanistic explanation by which EFNB2 affects endochondral ossification could be as follows. At the chondro-ossesous junction in the growth plates, osteoclast activity follows vascular invasion and is required for the conversion of cartilage to bone. The communication between chondrocytes and adjacent osteoclasts is critical for this process. Although RANKL/RANK signaling has been implicated, additional studies have demonstrated that there is an alternative pathway in which the insulin-like growth factor (IGF)-1/IGF-1 receptor (IGF-1R) signaling, a system well known to play a fundamental role during endochondral bone formation [37–39], regulates EFNB2 and its specific receptor EphB4. Hence, IGF-1 signaling induces EFNB2/EphB4 expression in osteoblasts and chondrocytes and EFNB2 expression in osteoclasts. This upregulation of EFNB2/EphB4 mediates cell-cell communication necessary for IGF-1 stimulation of osteoblast, osteoclast and chondrocyte differentiation required for endochondral bone formation [40]. In addition, during the endochondral bone formation, VEGF, among other factors, directs adjacent inner perichondrial cells to become osteoblasts and form the bone collar [41]. Interestingly, Wang et al. [42] showed that IGF-1-increased EFNB2 production stimulates VEGF expression and vascularization. Data from the present study demonstrate that in the absence of EFNB2, decreased VEGF is found in the hypertrophic zone of developing growth plate cartilage, reflecting, at least in part, reduced vascularization in this zone.

Vascularization of the un-mineralized transverse partition of cartilage columns is a critical step in endochondral bone formation; chondrocytes within cartilage terminally differentiate into hypertrophic chondrocytes, which produce VEGF to stimulate angiogenesis. Such an effect in EFNB2^Col2^KO mice was not surprising, as EFNB2 has been found to be expressed by hypertrophic chondrocytes in the developing growth plate [43], to play a crucial role in VEGF-induced angiogenesis [44, 45], to act as a pro-angiogenic factor in postnatal neovascularization, and to be involved in the formation of the primary blood capillary [9, 10, 46–48].

Ossification begins with invasion of the calcified hypertrophic cartilage by capillaries, but the remodeling of bone matrix by osteoclasts is also of major importance, as it results in a cavity filled with vascular channels containing hematopoietic cells. The newly formed blood vessels bring in osteoclast-like cells, which resorb the mineralized cartilage [49]. The decreased number of TRAP-positive cells and increase in mineralized cartilage in EFNB2^Col2^KO probably resulted from a delay in degradation of mineralized cartilage from the hypertrophic chondrocytes themselves [50] and chondroclasts and/or preosteclasts and osteoclasts, due to defective vascularization [43], culminating in altered bone growth. These findings suggest that EFNB2 acts on blood capillary invasion into hypertrophic chondrocytes, regulating angiogenesis at the chondro-osseous junction, thus facilitating endochondral bone development.

Endochondral ossification is a process that occurs from the embryonic stages through adulthood, permitting skeletal structures to be sustained during rapid bone growth. Our findings from imaging and bone densitometry showed that EFNB2^Col2^KO mice at P21 and 8 weeks of age displayed reduced bone mass with lower mineral content and trabeculae formation, resulting from the altered endochondral ossification, which impacted the long-term long bone development. However, although 1-year-old EFNB2^Col2^KO mice were smaller in size and length and had a significantly lower mineral content in the distal femur, other features such as pelvic size and whole body and vertebral mineral content did not differ from controls. An explanation for the latter observation could be that the independent, highly coordinated bone remodeling process [51, 52], which is responsible for removal and repair of damaged bone to maintain the integrity of the adult skeleton and mineral homeostasis, could have superseded the effect of the lack of EFNB2. Furthermore, several compensatory mechanisms that evolve during development could also compensate for the loss of EFNB2.

Importantly, cartilage-specific EFNB2 deletion also leads to spontaneous OA features, in which both cartilage and synovial membrane display alterations. However, the effect is not due to the abnormal gait of the cartilage-specific EFNB2^Col2^KO mice, as only 27 % demonstrated the locomotor phenotype, and the OA features were present in all mutant mice. Data from this study, in which the lack of EFNB2 led to OA, concur with and substantiate findings demonstrating that the presence and role of this factor in human chondrocytes positively impact the abnormal metabolism of cartilage in OA [12]. Moreover, this study also concurs with in vivo overexpression of the EFNB2 specific receptor, EphB4, in articular tissue, also demonstrating a significant decrease in progression of OA [15]. All these data thus suggest EFNB2 as an attractive therapeutic target in OA. As a continuation of this study, it would be of interest to explore whether, using this EFNB2^Col2^KO mouse model, surgically induced OA, such as the destabilization of the medial meniscus (DMM), would lead to development of more OA features.

Of the cartilage-specific EFNB2^Col2^KO mice 27 % displayed a locomotor phenotype, which occurred as soon as they learned to walk at P15 and continued throughout their lifespan. One could question the low or incomplete penetrance of the locomotor phenotype. Most explanations of incomplete penetrance in genetically identical individuals assumed differences in gene expression or somatic genetic or epigenetic variations [53, 54]. Recently, an additional model of a fundamentally different mechanism was suggested, involving stochastic cell behavior in the colonization process during embryonic development, which results in variable success in colonization, hence allowing for incomplete penetrance [55]. All these may arise stochastically, but once present, they drive the phenotype so that some individuals are affected while others with the same primary disease-causing mutation are not.

We speculated that this locomotor phenotype was due to abnormalities in hip development. However, for mice with this phenotype, although pelvic bone and canal width were smaller at 8 weeks, no differences were observed at 1 year of age. Furthermore, there were no differences in the acetabular parameters, suggesting that it did not result from any developmental hip abnormality. Yet, the walking defect in these EFNB2^Col2^KO mice resembled that which was previously demonstrated by two other members of the EFN family, specifically in mice lacking EFNB3 and its specific receptor EphA4 [28, 29]. For these EFN members, the defect was demonstrated to be related to a neurological defect occurring during the neurodevelopment in which there is a major disruption in the corticospinal tract, a pathway that sends messages from the brain to the moving limbs. How can this be related to our cartilage conditional KO model? The Col2a1 Cre promoter is reported to be expressed during early embryonic development (E9.5-11.5) on the notochord and developing brain [56] and at later stages in all cartilaginous tissues. As EFNB2 also plays an important role during neuronal development [57], in which it is found to guide boundary and synapse formation, cell migration and axon guidance, to name a few [4, 58–61], we therefore further explored the cause of the phenotype using a tracer of the corticospinal tract. The mice had an abnormal corticospinal projection pattern, with far more numerous ipsilateral projections in comparison to control mice. This finding is in line with reports on mice with EphA4 deletion [62] and EFNB3 deletion [29], which have similar behavioral defects. In brief, this pathway depends on the crossing of neurons from one side of the central nervous system to the other. The axons extending from neurons in the motor cortex of one hemisphere of the brain typically connect to targets on the opposite side of the spinal column. Therefore, the left side of the brain controls limb movement on the right side, and the right side of the brain controls limb movement on the left side. This result demonstrates the need for caution when the Col2a1 Cre promoter is used in transgenic mice. However, the vascular impairment, delayed mineralization and osteopenia displayed by the EFNB2^Col2^KO mice are clearly independent of the neurological defect found in these mice. From the present data, it cannot yet be determined whether the spontaneous OA phenotype observed during aging is secondary to the observed developmental defects or whether it is caused by an independent role of EFNB2 in adult articular cartilage. An in vivo study with a specific inactivation of the EFNB2 gene in adult articular cartilage using an inducible Cre system could resolve this issue.

## Conclusion

In summary, we showed for the first time that EFNB2 is required for normal endochondral ossification, cartilage/bone growth and development, and adult articular cartilage and synovial membrane preservation in vivo.

## Additional files

Additional file 1: Ephrin-B2 (EFNB2)^fl/fl^ mouse at 21 days old. EFNB2^fl/fl^ as control mouse, does not exhibit any locomotor phenotype. (MP4 3672 kb) Additional file 2: Ephrin-B2 (EFNB)2^Col2^ knockout (KO) mouse at 21 days old. EFNB2^Col2^KO mouse exhibits a locomotor phenotype. (MP4 4670 kb)
